# Basigin-2 is the predominant basigin isoform that promotes tumor cell migration and invasion and correlates with poor prognosis in epithelial ovarian cancer

**DOI:** 10.1186/1479-5876-11-92

**Published:** 2013-04-08

**Authors:** Shu-Hua Zhao, Yu Wang, Li Wen, Zhen-Bo Zhai, Zhen-Hua Ai, Nian-Ling Yao, Li Wang, Wen-Chao Liu, Bi-Liang Chen, Yu Li, Hong Yang

**Affiliations:** 1 Department of Obstetrics and Gynecology, Xijing Hospital, The Fourth Military Medical University, Xi’an 710032, China; 2 Department of Oncology, State Key Discipline of Cell Biology, Xijing Hospital, The Fourth Military Medical University, Xi’an 710032, China; 3 Department of Orthodontics, School of Stomatology, The Fourth Military Medical University, Xi’an 710032, China; 4 Department of gynecology, Shaanxi province cancer hospital, Xi’an 710032, China; 5 Cell Engineering Research Centre and Department of Cell Biology, State Key Laboratory of Cancer Biology, The Fourth Military Medical University, Xi’an 710032, China

**Keywords:** Ovarian cancer, Basigin-2, Prognosis, Survival, Migration, Invasion

## Abstract

**Background:**

Basigin, which has four isoforms, has been demonstrated to be involved in progression of various human cancers. The aim of this study was to examine the prognostic value of basigin-2 protein expression in epithelial ovarian cancer. Furthermore, the function of basigin-2 in ovarian cancer was further investigated in cell culture models.

**Methods:**

Immunohistochemistry staining was performed to investigate basigin-2 expression in a total of 146 ovarian tissue specimens. Kaplan Meier analysis and Cox proportional hazards model were applied to assess the relationship between basigin-2 and progression-free survival (PFS) and overall survival (OS). Real-time PCR, RT-PCR and western blot were used to explore basigin-2, basigin-3 and basigin-4 expression in ovarian cancer cell lines and tissues. To evaluate possible contributions of basigin-2 to MMP secretion and cell migration and invasion, the overexpression vectors pcDNA3.1-basigin-2 and basigin-2 siRNA were transfected into HO-8910 and HO-8910 PM cells respectively.

**Results:**

High basigin-2 expression was associated with lymph-vascular space involvement, lymph node metastasis and poor prognosis of epithelial ovarian cancer. Multivariate analyses indicated that basigin-2 positivity was an independent prognostic factor for PFS (*P* = 0.006) and OS (*P* = 0.019), respectively. Overexpression of basigin-2 increased the secretion of MMP-2/9 and cancer cell migration and invasion of HO-8910 cells, whereas knockdown of basigin-2 reduced active MMP-2/9 production, migration and invasion of HO-8910 PM cells.

**Conclusions:**

The expression of basigin-2 might be an independent prognostic marker and basigin-2 inhibition would be a potential strategy for epithelial ovarian cancer patients, especially in inhibiting and preventing cancer cell invasion and metastasis.

## Background

Ovarian cancer is the most lethal gynecologic malignancy among women in many countries [1]. Epithelial ovarian cancer is the most common ovarian cancer and accounts for approximately 70% of all ovarian malignant diseases. It is the sixth most common cancer and the fifth leading cause of cancer-related death in women worldwide [2]. Being largely asymptomatic, almost 70% of cases are at an advanced stage at diagnosis [3]. Despite significant advances in surgery and chemotherapy over the last few decades, therapeutic failure and disease progression are still quite frequent [4,5]. Therefore, there is an urgent requirement for new biomarkers for ovarian cancer, so as to develop better early diagnostic and therapeutic strategies.

Basigin, also known as CD147, extracellular matrix metalloproteinase inducer (EMMPRIN), neurothelin, 5A11, gp42, OX-47, and CE9, is a widely expressed integral plasma membrane glycoprotein that belongs to the immunoglobulin superfamily [6]. It is a highly glycosylated 58-kDa transmembrane protein that has four isoforms [7]. It turned out to be multifunctional and involved in various physiological processes such as fetal development, reproduction, T cell differentiation, and neural and retinal functions [6,8,9]. Increased expression of basigin has been found in several human tumors suggesting a role in human carcinogenesis. Moreover, it has been shown that higher expression of basigin was associated with poor prognosis of cancer patients and could serve as an independent predictor of poor survival in cancer [10-18]. The most life-threatening aspects of the oncogenic process are invasion and metastasis. Basigin has been shown to induce the production of several Matrix metalloproteinases (MMPs) which play an important role in tumor invasion and metastasis formation [19-32].

Basigin has four isoforms, namely basigin-1, basigin-2, basigin-3 and basigin-4. The basigin-1 transcript (NM_001728) is specific expressed on retina tissue and encodes a long three Ig-like domains in its extracellular portion [33]. The basigin-2 transcript (NM_198589) is the most predominant splice variant, encoding two Ig-like extracellular domains and being well-known as basigin/CD147/EMMPRIN [7]. Basigin-3 (NM_198590) and basigin-4 (NM_198591) are the other two short isoforms, encoding only one Ig-like domain in its extracellular portion, respectively. Basigin-4 was reported to overexpress in cervical cancer and promote proliferation of cervical cancer cell [34]. Our previous study showed Basigin-3 and Basigin-4 were up-regulated in hepatocellular carcinoma tissues and Basigin-3 exerted an inhibitory function to proliferation and invasion of HCC [35].

This is the first report that has examined the expression patterns of basigin and its isoforms in ovarian cancer tissues and cell lines. We also investigated the correlations between basigin-2 expression and various clinic pathologic parameters, and its prognostic value for survival of patients with epithelial ovarian cancer. Furthermore, we determined the functional roles of basigin-2 in ovarian cancer cell lines. The results presented here help to evaluate the suitability of basigin-2 as therapeutic target.

## Methods

### Patients and tissue samples

This study was approved by the Ethics Committee of the Fourth Military Medical University. For real-time PCR analysis, we collected data on 98 patients, including epithelial ovarian cancer (71 cases) and normal ovary (27 cases), treated at Xijing Hospital between November 2009 and June 2011. For immunohistochemical analysis, 146 patients with epithelial ovarian cancer who underwent surgical treatment at Xijing Hospital between May 2004 and July 2006 were included in this study. Patients whose cause of death remained unknown were excluded from our study. Ages of the 146 patients with ovarian carcinoma ranged from 26 to 79 years (mean, 52.8 years) and clinicopathological characteristics of the tumor sets are described in Table 1. Clinical data were obtained from clinical databases and tumors were staged according to International Federation of Gynecology and Obstetrics (FIGO) guidelines. None of the patients underwent chemotherapy or other adjuvant treatments before surgery. All patients received postoperative, platinum-based chemotherapy, but no radiotherapy. Informed consent was obtained from each patient before sample collection. The follow-up interval was calculated from the date of surgery to the date of death or last clinical evaluation. The follow-up time was from the date of surgery to March 2012, and the median time was 36 months (7 to 82 months). Eighty-five (58%) of these relapsed and 108 (74%) died during the course of the follow-up period. The duration of overall survival was measured from the date of diagnosis to death or censored at the date of last follow-up. Tumour progression was defined based on clinical, radiological or histological diagnosis.

**Table 1:** Relationship between basigin-2 expression with clinicopathological features of patients

**Characteristic**	**No.**	**Basigin-2 expression**		***P***
		**Low (n%)**	**High (n%)**	
Age				0.683
<60	95	34 (35.8)	61 (64.2)	
≥60	51	20 (39.2)	31 (60.8)	
FIGO stage				0.412
I+II	48	20 (41.7)	28 (58.3)	
III+IV	98	34 (34.7)	64 (65.3)	
Grade				0.478
G1	27	12 (44.4)	15 (55.6)	
G2	45	18 (40.0)	27 (60.0)	
G3	74	24 (32.4)	50 (67.6)	
Histological type				0.564
Serous	64	22 (34.4)	42 (65.6)	
Nonserous	82	32 (39.0)	50 (61.0)	
LVS involvement				0.012*
Negative	120	50 (41.7)	70 (58.3)	
Positive	26	4 (15.4)	22 (84.6)	
Lymph node metastasis				0.017*
Negative	111	47 (42.3)	64 (57.7)	
Positive	35	7 (20.0)	28 (80.0)	

*P < 0.05, LVS lymph-vascular space.

### Immunohistochemistry

Paraffin-embedded 4 μm-thick sections were deparaffinized, heated in citrate buffer (0.01 M), treated with 0.3% H_2_O_2_ (v/v), and re-hydrated. After blocking, the sections were incubated with basigin-2 antibody (HAb18, 1:200 dilution) in a humid chamber at room temperature for 1 h. HAb18, a monoclonal antibody against the Ig-C2 domain (specific to basigin-2), was produced and characterized in our laboratory [36,37]. The sections were rinsed and incubated for 30 min with the biotinylated second antibody. After washing, the slides were exposed to diaminobenzidine, and counterstained with hematoxylin. After serial dehydration, the slides were mounted for microscopic examination. Before staining the biopsies of selected patients, we optimized our staining procedure by comparing different antigen retrieval methods and testing different antibody dilutions in epithelial ovarian cancer biopsies. As positive controls epithelial ovarian cancer tissue that showed positive staining in earlier staining procedures was used. As negative control for the staining procedure, the primary antibody was omitted. The intensity of basigin-2 staining was scored as 0 (no signal), 1 (weak), 2 (moderate), and 3 (marked). Percentage scores were assigned as 1, 1-25%; 2, 26-50%; 3, 51-75%; and 4, 76-100%. The scores of each tumor sample were multiplied to give a final score of 0–12, and the tumors were finally determined as negative (−), score 0; lower expression (+), score ≤4; moderate expression (++), score 5–8; and high expression (+++), score ≥9. In this study, we grouped all of the samples into the high expression group (++ or +++) and the low expression group (− or +) according to the protein expression. Two of the pathologists, without prior knowledge of the clinical data, independently graded the staining intensity in all cases.

### Cell lines

3-AO, SKOV-3, HO-8910 and HO-8910 PM (a highly metastatic cell line derived from HO-8910) ovarian cancer cells were purchased from the Cell Bank of Type Culture Collection of the Chinese Academy of Sciences (Shanghai, China) [38]. A-2780 cells were purchased from the American Type Culture Collection. All cell lines were cultured using standard protocols.

### Cell culture and transfection

The cells were maintained at 37°C under 5% CO_2_ in DMEM containing 10% (v/v) FBS. The coding regions of basigin-2 were inserted into pcDNA3.1 (Clontech). We transfected the plasmids into the cells using Lipofectamine 2000™ reagent (Invitrogen, USA) and incubated the cells for 48 h before analysis. The expression of basigin-2 protein was confirmed by western blot analysis using HAb18.

### Gene silencing using siRNA

HO-8910 PM cells were transfected with basigin-2 siRNA by the Lipofectamine™ 2000 (Invitrogen, USA) according to the manufacturer’s instructions. A nonspecific control was used as non-targeting siRNAs. Twenty-four hours after transfection, transfected cells were examined for gene deletion. The sequences of oligos are as follows; sense siRNA: 5^′^-UGUUCGUGCUGCUGGGAUUTT-3^′^, and antisense siRNA: 5^′^-AAUCCCAGCAGCACGAACATT-3^′^.

### Gelatin zymography

To determine the enzyme activities of MMP-2 and MMP-9, media derived from the ovarian cancer cells cultured for 48 hours were used for the gelatin zymography. Culture media were subjected to a 10% SDS-PAGE, in which 1 mg/ml gelatin (type A from porcine skin) had been incorporated. After electrophoresis, the gels were washed for 1 h in a buffer containing 2.5% Triton X-100 and then incubated overnight in a digestion buffer containing calcium and zinc at 37°C with constant stirring for 24 h. Then, gels were stained with 0.25% Coomassie Brilliant Blue R-250 (Sigma, USA) and distained in 7.5% acetic acid with 20% methanol. MMP-2 and MMP-9 activities were quantified by densitometry using Quantity One software.

### RT-PCR

Total RNA was harvested from cultured cells or tissues using the RNeasy minikit (Qiagen, Germany) and reverse transcribed into cDNA using the PrimeScript reverse transcription-PCR (RT-PCR) kit (TaKaRa, Japan). PCRs were performed using Ex *Taq* HS (TaKaRa) with the following program: 94°C for 2 min; 30 cycles of 94°C for 30 s, 56°C for 30 s, and 72°C for 60 s; and a final step of 10 min at 72°C. PCR products were analyzed using agarose gel electrophoresis and cloned into the pMD18-T (TaKaRa) vector for sequencing. The following primers were used: for basigin-2, 5^′^-ATGGCGGCTGCGCTGTTCGTGCTG-3^′^ (forward); 5^′^-GGCCTCCATGTTCAGGTTCTCAAT-3^′^ (reverse); for basigin-3 and basigin-4, 5^′^-GGCTTAGTCTGCGGTCCTCTTGCA-3^′^ (forward); 5^′^-GGCCTCCATGTTCAGGTTCTCAAT-3^′^ (reverse); for β-actin, 5^′^-CGGGACCTGACTGACTACCTC-3^′^ (forward); 5^′^-TCGTCATACTCCTGCTTGCTG-3^′^ (reverse).

### Real-time quantitative RT-PCR

TaqMan primer and probe pairs specific to basigin variants and β-actin were synthesized by Invitrogen (Shanghai, China). Real-time quantitative RT-PCR was performed using the Stratagene Mx3005p multiplex quantitative PCR system (La Jolla, CA) and Premix Ex *Taq* reagent (TaKaRa). The cycling program was 94°C for 2 min and 40 cycles of 94°C for 15 s, 58°C for 20 s, and 72°C for 20 s. The results were normalized as the ratio of absolute mRNA copy numbers of basigin variants to those of β-actin, which were calculated from standard curves that were obtained from dilutions of precisely quantified template plasmid. The following primers were used: for basigin-2, 5^′^-TCGCGCTGCTGGGCACC-3^′^ (forward); 5^′^-TGGCGCTGTCATTCAAGGA-3^′^ (reverse), 5^′^FAM-CCGGGGCTGCCGGCACAGTC-TAMRA3^′^ (TaqMan probe); for basigin-3, 5^′^-GACGCGTCTCCCCAAGAAA-3^′^ (forward); 5^′^-GAGCAGGTGAGGAGTATCTTGGA-3^′^ (reverse), 5^′^FAM-CCGGCACAGTCTTCACTACCGTAGAAGACCT-TAMRA3^′^ (TaqMan probe); for basigin-4, 5^′^-GTTGCTGAGAGTCTGGGTTTACG-3^′^ (forward); 5^′^-GGGAGGAAGACGCAGGAGTAC-3^′^ (reverse), 5^′^ FAM-CCAAGAAAGGGTGGACTCCGACGACC-TAMRA 3^′^ (TaqMan probe); for β-actin, 5^′^-CCCAGCCATGTACGTTGCTA-3^′^ (forward); 5^′^-TCACCGGAGTCCATCACGAT-3^′^ (reverse), 5^′^FAM-CGCCTCTGGCCGTACCACTG-TAMRA3^′^ (TaqMan probe).

### Western blot analysis

Cells were lysed in lysis buffer containing protease inhibitor cocktail. Protein concentration was determined using a BCA protein assay kit (Pierce, USA). Equal amounts of protein lysate were electrophoretically separated on 10% sodium dodecyl sulfate-polyacrylamide gels and transferred to PVDF membranes (Millipore, USA). The membranes were blocked with 1% BSA for 2 h at room temperature and incubated with HAb18 and β-tubulin (Santa Cruz, USA), followed by horseradish peroxidase-conjugated secondary antibody. Protein expression levels were quantified using the software Quantity One to detect intensity of the protein bands.

### Wound-healing assay

Cell migration was measured by the in vitro scratch wound-healing assay. Ovarian cancer cells were incubated in 24-well plates and a small wound area was made in the confluent monolayer with a scraper. Then, the cells were rinsed two times with PBS and fresh culture medium was added. Wound closure was monitored at various time points by observation under a microscope, and the degree of cell migration was quantified by the ratio of gap distance at 36 h to that at 0 h. Experiment was repeated three times.

### Cell migration and invasion assays

The invasive and migratory potential of cells was evaluated using Transwell chambers with 8 μm pores (BD Biosciences, USA). For cell invasion assays, after 24 h transfection, 3.0 × 10^5^ cells in serum free medium were added to each upper insert pre-coated with matrigel matrix (BD Biosciences, USA). The bottom chamber contained standard medium with 10% FBS. After 48 h incubation, non-invasion cells on the upper surface of the filters were removed gently by a cotton-tip swab, and invaded cells on the lower membrane surface were fixed in methanol, stained with 0.1% crystal violet, photographed, and counted. Cell migration assay was performed according to the protocol described above, except that the cells were added into the inserts with 24-hour incubation without matrix gel pre-coated. Each experiment was carried out in triplicate wells and repeated at least three times.

### Statistical analysis

The correlations between immunohistochemical expression and the clinical variables were evaluated by the χ^2^-test or Fisher’s exact test, as appropriate. PFS and OS curves were calculated with the Kaplan-Meier method and were analyzed with the log-rank test. Cox proportional hazards modeling of factors potentially related to survival was performed to identify factors that might have a significant influence on survival. One-way ANOVA was used to compare the MMP production, transfilter cell number, fluorescence signals and grayscale value in different groups. *P* < 0.05 was considered statistically significant. All analyses were performed with SPSS 12.0 for Windows.

## Results

### Clinical significance of basigin-2 expression in epithelial ovarian cancer

**Figure 1:**
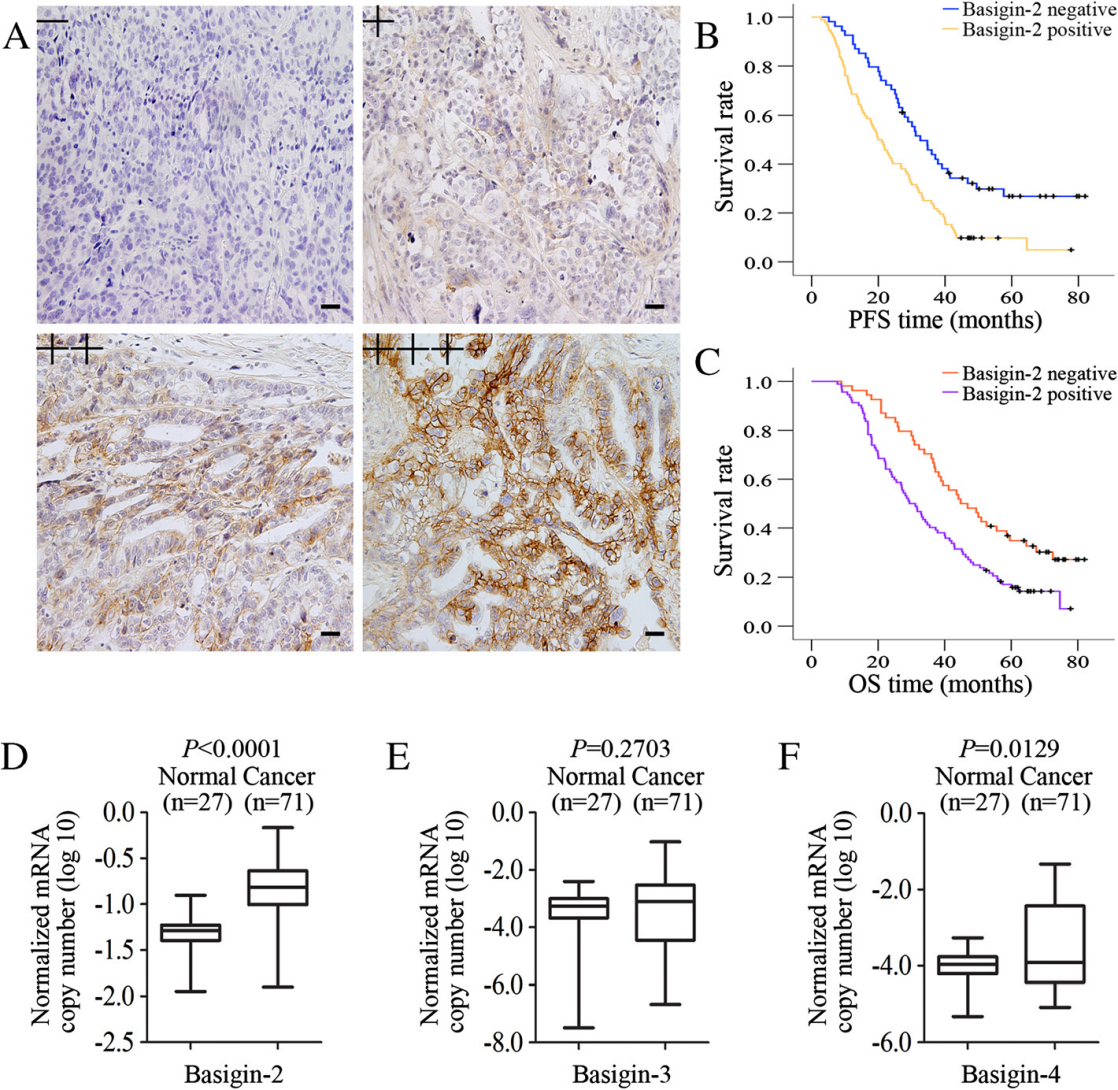
**Basigin-2 expression level and its prognostic effects in epithelial ovarian cancer. A** Representative images of Basigin-2 protein expression in paraffin-embedded tissue from 146 patients with epithelial ovarian cancer (−, +, ++, +++). Scale bars = 0.1 mm. **B** The progression-free survival curves for the high-basigin-2 expression group (n = 92) and the low-basigin-2 expression group (n = 54). The progression-free survival rates between the two groups showed significantly different (*P* < 0.001). **C** The overall survival curves for the high-basigin-2 expression group (n = 92) and the low-basigin-2 expression group (n = 54). The overall survival rates between the two groups showed significantly different (*P* = 0.001). **D-F** Overexpression of basigin variants in epithelial ovarian cancer tissues. The expression levels of basigin variants were calculated as the log10 value of the normalized ratio.

We examined the basigin-2 expression in epithelial ovarian cancer by immunohistochemical staining, using a total of 146 surgical specimens. As shown in Figure 1A, the immunoreactivity of basigin-2 was detected at variable levels, and was localised in the cytomembrane of tumor cells. The correlation of basigin-2 expression with clinicopathological features was shown in Table 1. Basigin-2 expression was positively correlated with lymph-vascular space involvement (*P* = 0.012), lymph node metastasis (*P* = 0.017), but not with age, FIGO stage, degree of differentiation, or Histological type.

The each of PFS and OS curves calculated using the Kaplan-Meier method according to basigin expression was shown in Figure 1B and 1C (progression-free time, *P* < 0.001, Figure 1B; overall survival time, *P* = 0.001, Figure 1C). By using univariate Cox proportional analysis, basigin-2 expression was statistically correlated to progression-free survival (*P* < 0.001, Table 2) and overall survival (*P* = 0.002, Table 2). By using multivariate Cox proportional analysis, considering statistically significant variables and basigin-2 expression and FIGO staging were independent factors of progression-free and overall survival prognosis predictions (Tables 2 and 3).

**Table 2:** Univariate and multivariate analyses of PFS in patients with epithelial ovarian cancer

**Variables**	**Categories**	**Univariate analysis**		***P***	**Multivariate analysis**		***P***
		**HR**^**a**^	**95% CI**^**b**^		**HR**^**a**^	**95% CI**^**b**^	
Age	<60						
	≥60	1.131	0.779-1.640	0.518	–	–	–
FIGO stage	I+II						
	III+IV	1.882	1.261-2.809	0.002*	1.710	1.144-2.557	0.009*
Grade	G1						
	G2	1.171	0.686-1.997	0.563	–	–	–
	G3	1.419	0.864-2.330	0.166	–	–	–
Histological type	Serous						
	Nonserous	0.852	0.596-1.218	0.380	–	–	–
LVS involvement	Negative						
	Positive	1.944	1.246-3.033	0.003*	1.410	0.935-2.127	0.101
Lymph node metastasis	Negative						
	Positive	1.638	1.091-2.461	0.017*	1.578	1.001-2.488	0.050
Basigin-2	Low						
	High	2.046	1.388-3.017	<0.001*	1.758	1.179-2.619	0.006*

PFS progression-free survival, ^a^HR = hazard ratio, ^b^CI = confidence interval, *P < 0.05, LVS lymph-vascular space.

**Table 3:** Univariate and multivariate analyses of OS in patients with epithelial ovarian cancer

**Variables**	**Categories**	**Univariate analysis**		***P***	**Multivariate analysis**		***P***
		**HR**^**a**^	**95% CI**^**b**^		**HR**^**a**^	**95% CI**^**b**^	
Age	<60						
	≥60	1.202	0.825-1.752	0.338	-	-	-
FIGO stage	I+II						
	III+IV	1.806	1.202-2.713	0.004*	1.641	1.089-2.471	0.018*
Grade	G1						
	G2	1.154	0.677-1.969	0.598	-	-	-
	G3	1.282	0.776-2.116	0.332	-	-	-
Histological type	Serous						
	Nonserous	0.878	0.610-1.265	0.485	-	-	-
LVS involvement	Negative						
	Positive	1.696	1.126-2.555	0.012*	1.475	0.974-2.232	0.066
Lymph node metastasis	Negative						
	Positive	1.858	1.181-2.924	0.007*	1.530	0.961-2.435	0.073
Basigin-2	Low						
	High	1.882	1.272-2.785	0.002*	1.622	1.084-2.429	0.019*

OS overall survival, ^a^HR = hazard ratio, ^b^CI = confidence interval, *P < 0.05, LVS lymph-vascular space.

### Differential expression of basigin mRNA variants in ovarian normal and cancer tissues

27 normal and 71 tumor samples were used to analyze basigin-2, basigin-3 and basigin-4 mRNA expression by real-time quantitative PCR assay (Figure 1D-F). Expression level was shown as a ratio between basigin-2, basigin-3 and basigin-4, the reference gene β-actin to correct for the variation in the amounts of RNA. The results indicated that mRNA levels of basigin-2 and basigin-4 were significantly different between the cancer and normal samples. Expressions of basigin-2 and basigin-4 mRNAs in ovarian normal tissues were lower than that in cancer tissues (*P* < 0.001 and *P* = 0.0129, respectively), whereas mRNA level of basigin-3 showed no significant difference between ovarian normal and cancer tissues (*P* = 0.2703), as shown in Figure 1D-F.

### Basigin-2 protein expression in ovarian cancer cell lines

**Figure 2:**
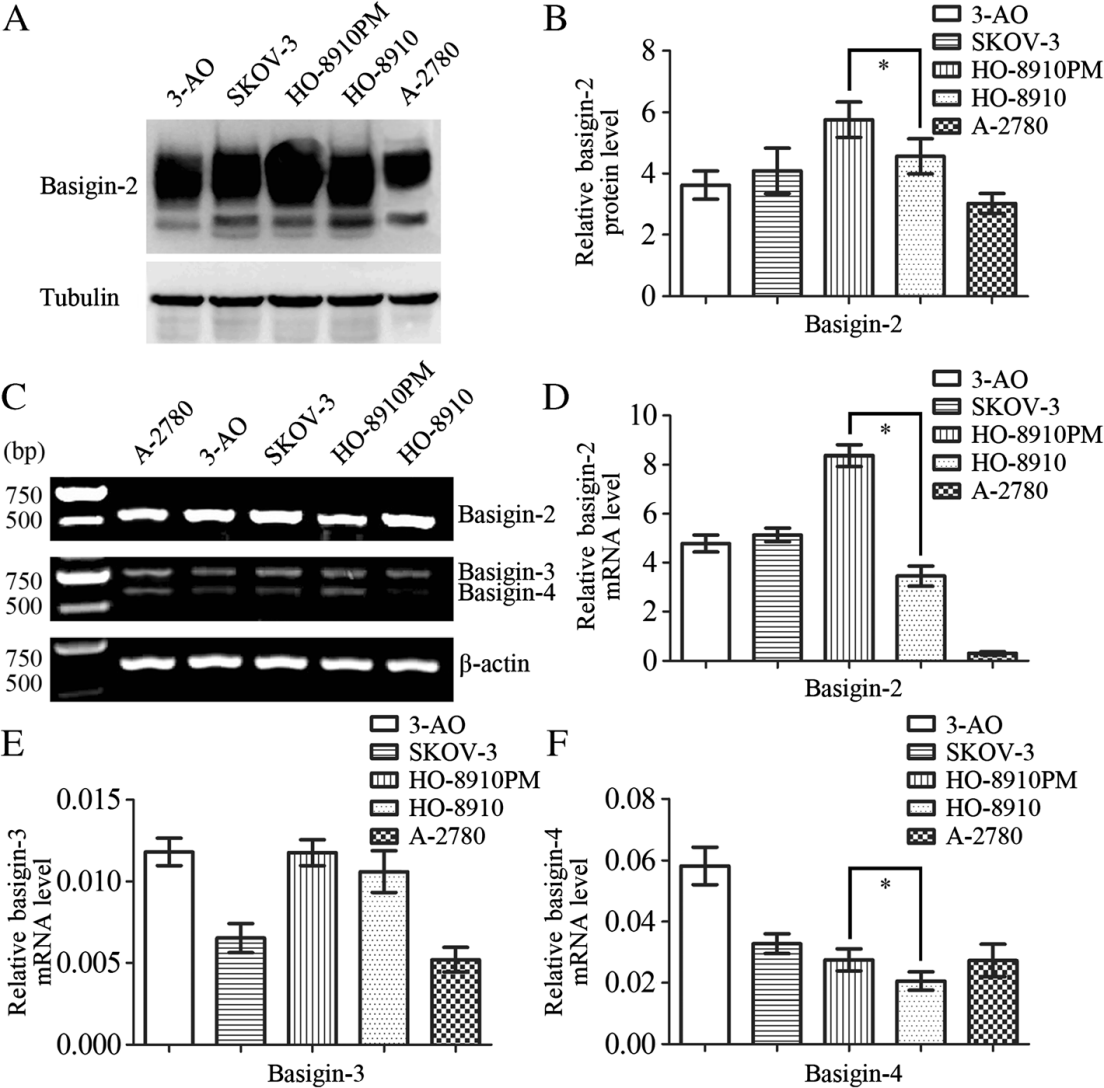
**Expression levels of basigin-2, basigin-3 and basigin-4 in ovarian cancer cells. A-B** Western blot analysis determining the relative protein expression levels of basigin-2 in ovarian cancer cells. Basigin-2 protein expression levels were quantified using Quantity One normalized against tubulin levels. **C** RT-PCR analysis of basigin-2, basigin-3 and basigin-4 mRNA expression levels in ovarian cancer cells. **D-F** Real-time PCR analysis determining the relative mRNA expression levels of basigin-2, basigin-3 and basigin-4 in ovarian cancer cells. Columns mean derived from at least three independent experiments, bars, SD. Statistical analysis was done using Student’s t tests. *P < 0.05, significant.

We used western blot analysis to characterize protein expression of basigin-2 in five ovarian cancer cell lines. Basigin-2 was detectable in all these five cell lines, with highest expression in HO-8910 PM cells. Expression of basigin-2 protein in HO-8910 cell line was lower than that in its subline cell line HO-8910 PM (a highly metastatic cell line derived from HO-8910), as shown in Figure 2A and 2B.

### Basigin-2, Basigin-3 and Basigin-4 mRNA expression in ovarian cancer cell lines

RT-PCR was performed to determine whether basigin mRNA variants were expressed in ovarian cancer cell lines. The results indicated that basigin-2, basigin-3 and basigin-4 were detectable in all these five cell lines (Figure 2C). We used real-time PCR analysis to characterize mRNA expression of basigin-2, basigin-3 and basigin-4 in five ovarian cancer cell lines. Expression of basigin-2 and basigin-4 mRNA in HO-8910 cell line was lower than that in its subline cell line HO-8910 PM, as shown in Figure 2D and 2F (*P* < 0.05). Expression of basigin-3 mRNA showed no significant difference between HO-8910 cells and HO-8910PM cells (*P* > 0.05).

### Effects on cellular invasiveness/migration and MMP-9/MMP-2 expressions of HO-8910 cells after transfection with basigin-2

**Figure 3:**
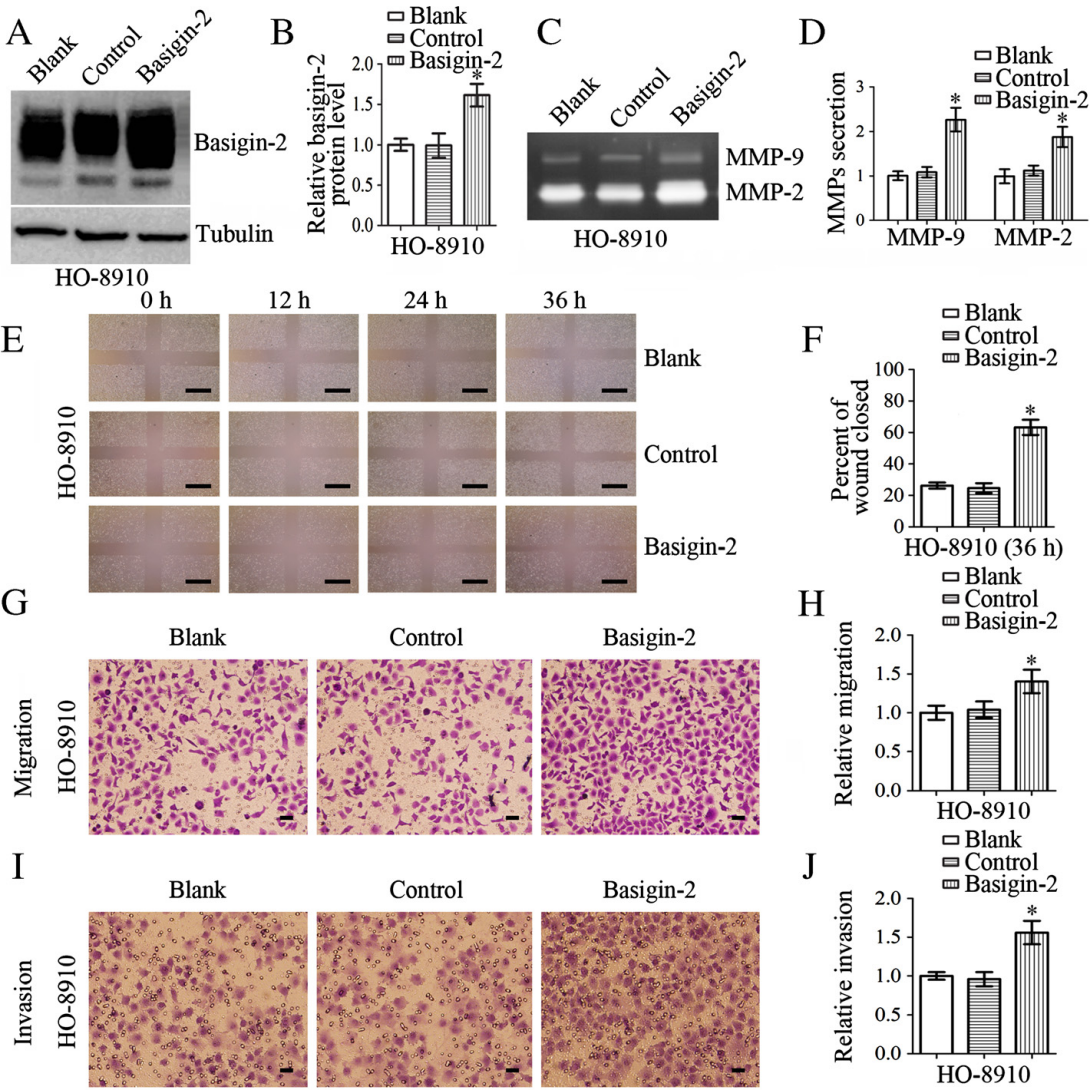
**Effects on cellular invasiveness/migration and MMP-9/MMP-2 expressions of HO-8910 cells after transfection with basigin-2.** HO-8910 cells were either untransfected (blank), or transfected with pcDNA3.1 (control) or pcDNA3.1-basigin-2 (basigin-2). **A-B** Western blot demonstrated basigin-2 protein levels were significantly increased after transfection with basigin-2. **C-D** HO-8910 cells were incubated for 24 h and conditioned media were used for the measurement of MMP-2 and MMP-9 protein levels by gelatin zymography. Zymographic analysis showed increased MMP-2 and MMP-9 activity in basigin-2-transfected HO-8910 cells. **E-F** Wound healing assay was performed on monolayers of HO-8910 cells transfected with pcDNA3.1-basigin-2. The wound gaps were measured at six reference points along the wound, and the results were expressed as the average wound gap. **G-H** Overexpression of basigin-2 in HO-8910 cells resulted in an increase in cellular migrate capacity. **I-J** Overexpression of basigin-2 in HO-8910 cells resulted in an increase in cellular invasive capacity. Scale bars = 0.5 mm (**E**). Scale bars = 0.1 mm (**G, I**). Statistical significance was assessed using one-way ANOVA. Data are presented as mean ± SD for three independent experiments. *P < 0.05, statistically significant difference.

HO-8910 cells were either untransfected (blank), or transfected with pcDNA3.1 (control) or pcDNA3.1-basigin-2 (basigin-2), as shown in Figure 3. After transfection of cells, western blot analysis was performed to analyze the expression levels of basigin-2 protein. As shown in Figure 3A and Figure 3B, the expression of basigin-2 protein was increased in pcDNA3.1-basigin-2-transfected cells compared with pcDNA3.1-transfected cells and untransfected cells (*P* < 0.05). Next, we employed gelatin zymography to analyze the effect of basigin-2 overexpression on MMP-2 and MMP-9 enzyme activities. We observed that MMP-2 and MMP-9 gelatinase activities in the culture media of pcDNA3.1-basigin-2-transfected HO-8910 cells were substantially higher than in cells transfected with control cells (Figure 3C and Figure 3D, *P* < 0.05).

Cellular invasiveness and migration were analyzed by both Transwell and wound healing assays. HO-8910 cells that were transfected with the pcDNA3.1-basigin-2 plasmid exhibited a significant increase in cellular migration as compared with control cells (Figure 3E-3H). Invasion is another important step for metastasis. To investigate the effects of pcDNA3.1-basigin-2 transfection on cell invasion, we determined the ability of pcDNA3.1-basigin-2-transfected cells to invade through Matrigel in Transwell chambers. As shown in Figure 3I and 3J, HO-8910 cells transfected with pcDNA3.1-basigin-2 displayed much higher invasive capability compared with control cells as evidenced by increased number of cells invading through the matrigel.

### Effects on cellular invasiveness/migration and MMP-9/MMP-2 expressions of HO-8910PM cells after transfection with basigin-2 siRNA

**Figure 4:**
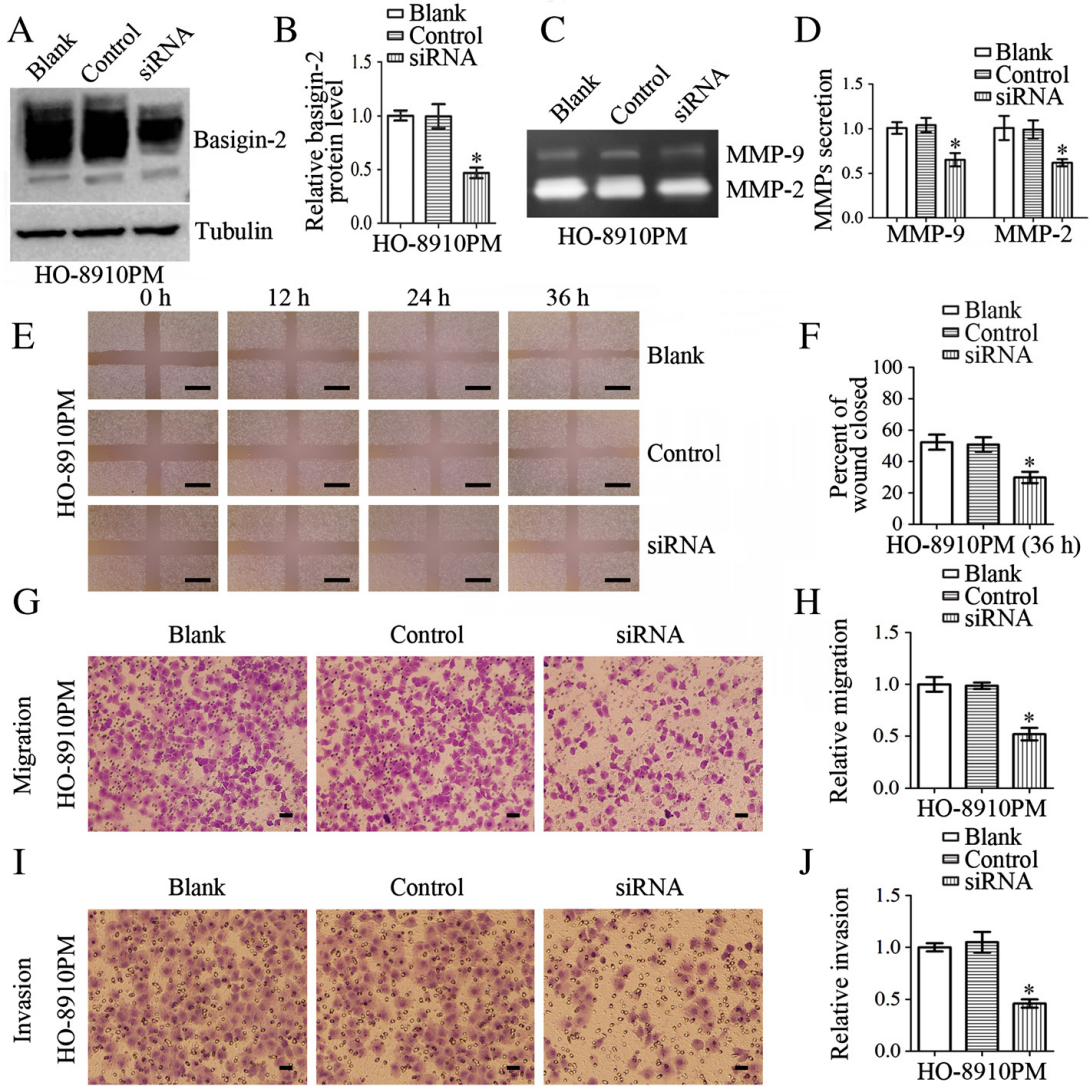
**Effects on cellular invasiveness/migration and MMP-9/MMP-2 expressions of HO-8910PM cells after transfection with basigin-2 siRNA.** HO-8910PM cells were either untransfected (blank), or transfected with nonspecific siRNA (control) or basigin-2 siRNA (siRNA). **A-B** Western blot demonstrated basigin-2 protein levels were significantly inhibited by basigin-2 siRNA. **C-D** HO-8910PM cells were incubated for 24 h and conditioned media were used for the measurement of MMP-2 and MMP-9 protein levels by gelatin zymography. Zymographic analysis showed decreased MMP-2 and MMP-9 activity in HO-8910PM cells transfected with basigin-2 siRNA. **E-F** Wound healing assay was performed on monolayers of HO-8910PM cells transfected with basigin-2 siRNA. The wound gaps were measured at six reference points along the wound, and the results were expressed as the average wound gap. **G-H** Suppression of basigin-2 expression in HO-8910PM cells resulted in significant reduction in cellular migration. **I-J** Suppression of basigin-2 expression in HO-8910PM cells resulted in significant reduction in cellular invasiveness. Scale bars = 0.5 mm (**E**). Scale bars = 0.1 mm (**G**, **I**). Statistical significance was assessed using one-way ANOVA. Data are presented as mean ± SD for three independent experiments. *P < 0.05, statistically significant difference.

HO-8910PM cells were either untransfected (blank), or transfected with nonspecific siRNA (control) or basigin-2 siRNA (siRNA), as shown in Figure 4. After transfection of cells, western blot analysis was performed to analyze the expression levels of basigin-2 protein. As shown in Figure 4A and Figure 4B, the expression of basigin-2 protein was decreased in basigin-2 siRNA-transfected cells compared with control siRNA-transfected cells and untransfected cells (*P* < 0.05). Next, we employed gelatin zymography to analyze the effect of silencing basigin-2 on MMP-2 and MMP-9 enzyme activities. We observed that MMP-2 and MMP-9 gelatinase activities in the culture media of basigin-2 siRNA-transfected HO-8910PM cells were substantially lower than in cells transfected with control cells (Figure 4C and Figure 4D, *P* < 0.05).

Cellular invasiveness and migration were analyzed by both Transwell and wound healing assays. The results showed a significant reduction in motility of basigin-2 siRNA-transfected cells compared with the control groups (Figure 4E-4H). Invasion is another important step for metastasis. To investigate the effects of basigin-2 siRNA transfection on cell invasion, we determined the ability of basigin-2 siRNA-transfected cells to invade through Matrigel in Transwell chambers. As shown in Figure 4I and 4J, a significant reduction in the number of invading cells was observed for siRNA-transfected cells after 24 h compared with the control cells.

## Discussion

Previous studies have shown that basigin is one of the most commonly up-regulated genes in human tumors. Importantly, its expression is often correlated with tumor metastasis and poor prognosis [10-18]. There are already papers showing that basigin is associated with the survival and progression of ovarian cancer, and is considered as a biomarker of poor outcome [39-41]. However, some results show that basigin is a significant indicator of a favorable prognosis of ovarian cancer [42,43]. In the present study, we described the relationship between basigin-2 expression and clinicopathological parameters of 146 cases of epithelial ovarian cancer with immunohistochemical analysis. Our results indicated that high basigin-2 expression was significantly correlated with lymph-vascular space involvement and lymph node metastasis, suggesting that its expression might be important for the acquirement of malignant potential in epithelial ovarian cancer. Moreover, high basigin-2 expression was associated with significantly shorter overall survival and progression-free survival in epithelial ovarian cancer. We evaluated prognostic value of various factors by univariate and multivariant COX proportional analysis, and found that basigin-2 expression was significantly associated with both overall survival and progression-free survival. These results led us to believe that basigin-2 might be used as a candidate biomarker for epithelial ovarian cancer diagnosis and therapy.

As far as we know, this is the first report that we checked basigin variants expression level in ovarian cancer tissues and cell lines. In the present study, we showed that basigin-2 and basigin-4 mRNA expressions determined by real-time quantitative PCR were significantly higher in ovarian cancer tissues than in normal ovarian tissues. We also showed that basigin-2, basigin-3 and basigin-4 were detectable by real-time quantitative PCR assays in ovarian cancer cell lines. We found basigin-2 and basigin-4 are up-regulated in high metastasis ovarian cancer cell line HO-8910PM comparing to its parent cell line HO-8910. Cause mRNA level of basigin-2 was much higher than basigin-4 in ovarian cancer (>100 fold to basigin-4), we focused our further study on basigin-2. Diagnosis of epithelial ovarian cancer usually occurs when the cancer has already progressed to the advanced stages [3]. Invasion and metastasis are characteristic features and the main factors related to the poor prognosis in patients with epithelial ovarian cancer. Therefore, it is important to understand the molecular mechanisms involved in the pathogenesis and progression of metastasis to the development of novel therapies to treat epithelial ovarian cancer. The major mediators of basement membrane degradation are the matrix metalloproteinases (MMPs) [26,44]. The MMPs, a family of zinc-dependent endopeptidases, play a prominent role in in extracellular matrix degradation associated with cancer cell invasion and metastasis [45-48]. MMP-2 and MMP-9, in particular, have been found to be specifically associated with ovarian cancer metastasis [49,50]. Basigin has been shown to promote the invasion and migration of tumor cells by induction of MMP activities which is regulated by basigin isoforms and glycosylation patterns [35,51]. In order to further investigate the effects of basigin-2 on ovarian cancer progression, pcDNA3.1-basigin-2 or basigin-2 specific siRNA was transfected into HO-8910 cells or HO-8910PM cells, respectively. In vitro examinations revealed that basigin-2 overexpression was associated with increased enzymatic activity of MMP-2 and MMP-9, resulting in increased invasiveness and migration in HO-8910 cells. Furthermore, our results demonstrated that knockdown of basigin-2 expression by siRNA impaired the migratory and invasive abilities and down-regulated the activities of both MMP-2 and MMP-9 in HO-8910PM cells. These data are consistent with previous studies [19-32]. Induction of MMPs activity by basigin is regulated by isoform and glycosylation pattern. We have previously shown that basigin-3 was one of the most important isoforms of the basigin family, and it inhibited hepatocellular carcinoma cell proliferation, MMP induction, and cell invasion, probably via hetero-oligomerization with basigin-2 [35]. However, expression pattern of basigin is different in ovarian cancer. Basigin-4, not basigin-3 was found to over-express in ovarian tissues besides basigin-2 and may play more important role on metastasis of ovarian cancer than basigin-3 cause its higher expression in HO-8910PM cells. Taken together, overexpression of basigin-2 increased the secretion of MMP-2/MMP-9 and cancer cell migration and invasion of HO-8910 cells, whereas the inhibition caused by basigin-2 siRNA affected HO-8910PM cell migration and invasion through MMP-2 and MMP-9 expression. These data suggest that the suppression of basigin-2 expression has potential for antimetastatic therapy by inhibiting activity of MMP-2 and MMP-9.

## Conclusions

In summary, this study is the first to examine the expression patterns of basigin and its isoforms in ovarian cancer tissues and cell lines. Our study demonstrated that the expression level of basigin-2 was highly increased in epithelial ovarian cancer. Moreover, basigin-2 protein expression was significantly correlated with survival and the expression level of MMP-2/MMP-9 and malignant metastasis of ovarian cancer. This study further elucidates the molecular mechanisms underlying invasion and metastasis of ovarian cancer. These data suggest that basigin-2 may serve as a promising therapeutic target for ovarian cancer and that down-regulating basigin-2 could potentially be an effective therapeutic approach for inhibition of ovarian cancer progression.

## Abbreviations

MMP-2: Matrix metalloproteinase-2; MMP-9: Matrix metalloproteinase-9; PFS: Progression-free survival; OS: Overall survival; FIGO: International federation of gynecology and obstetrics; HCC: Hepatocellular caicinoma; AS: Alternative splicing.

## Competing interests

The authors declare that they have no competing interests.

## Authors’ contributions

HY, BLC, WCL and YL are responsible for the study design. SHZ, YW, and ZBZ performed the experiments and draft the manuscript. SHZ, ZHA and NLY collected the data. YW, SHZ, LW^3^, and LW^5^ participated in the data analysis and interpretation. All authors read and approved the final manuscript.
